# Optimization of robotic path planning and navigation point configuration based on convolutional neural networks

**DOI:** 10.3389/fnbot.2024.1406658

**Published:** 2024-06-04

**Authors:** Jian Wu, Huan Li, Bangjie Li, Xiaolong Zheng, Daqiao Zhang

**Affiliations:** ^1^Xi'an Institute of High-Tech, Xi'an, China; ^2^School of Software, Xinjiang University, Urumqi, China

**Keywords:** robotic path planning, precise area coverage, optimized point configuration, convolutional neural networks, navigation

## Abstract

This study introduces a novel approach for enhancing robotic path planning and navigation by optimizing point configuration through convolutional neural networks (CNNs). Faced with the challenge of precise area coverage and the inefficiency of traditional traversal and intelligent algorithms (e.g., genetic algorithms, particle swarm optimization) in point layout, we proposed a CNN-based optimization model. This model not only tackles the issues of speed and accuracy in point configuration with Gaussian distribution characteristics but also significantly improves the robot's capability to efficiently navigate and cover designated areas with high precision. Our methodology begins with defining a coverage index, followed by an optimization model that integrates polygon image features with the variability of Gaussian distribution. The proposed CNN model is trained with datasets generated from systematic point configurations, which then predicts optimal layouts for enhanced navigation. Our method achieves an experimental result error of <8% on the test dataset. The results validate effectiveness of the proposed model in achieving efficient and accurate path planning for robotic systems.

## 1 Introduction

In the domain of robotic path planning and navigation, the strategic configuration of points, particularly those with Gaussian distribution characteristics within polygonal areas, is fundamental to enhancing efficiency and precision in covering specific regions (Chao et al., 2023; Cui et al., 2023; Wu X. et al., 2023). The concept of coverage fraction is pivotal in evaluating the effectiveness of these configurations. Due to the inherent randomness and the possibility of overlapping point coverage, the direct derivation of analytical models for calculating the coverage fraction proves to be a formidable challenge. This complexity necessitates a shift toward discretization, transforming the continuous challenge into a discrete problem that can be methodically approximated. Traditional methods, such as sequential traversal, while methodical, are markedly inefficient and unable to satisfy the exigencies of rapid, real-time decision-making essential in autonomous navigation. Lu et al. (2019) and Jie et al. (2023) reveal attempts to address these limitations, with genetic algorithms and particle swarm optimization offering efficiency improvements.

However, these methods still suffer from significant drawbacks, including sensitivity to computation times and unpredictability in outcomes, which hinder their applicability in scenarios demanding high precision and responsiveness. Addressing these challenges, our research plays a pivotal role in the exploitation of deep learning networks (Simsekli et al., 2019), which is renowned for their robust learning capacities and adaptability. This study unfolds in progressive stages: it commences with establishing an optimal point configuration algorithm derived from the traversal methodology (Luo et al., 2020; Hu et al., 2021; Heßler and Irnich, 2022). It then advances to devising a method for generating random polygons, analyzing the elements that impact traversal searches, and determining the optimal traversal strides for generating models for optimal point configuration. The cornerstone of our approach is the application of feature extraction and dimensionality reduction techniques on the conditions triggering configuration and facilitating the creation of a convolutional neural network (CNN)-based model for point configuration (Jing et al., 2022). This model is meticulously trained on a dataset designed to reflect various polygon shapes and configurations, paving the way for an innovative approach to path planning and navigation. Our findings, which are supported by simulation and practical implementation, demonstrate the model's unparalleled effectiveness and efficiency. The CNN-based approach significantly outperforms the traversal engineering algorithm, genetic algorithm, and particle swarm optimization (Langazane and Saha, 2022; Aote et al., 2023) in terms of speed, accuracy, and real-time adaptability, heralding a new era in robotic navigation. By optimizing point configuration through deep learning, robots can now navigate and cover specific areas with unprecedented precision, marking a milestone in the quest for advanced robotic path planning and navigation solutions. This introduction sets the stage for a detailed exploration of our methodology, the neural network model, and the profound implications of our study in the broader context of robotics and autonomous systems. The contributions of this study are as follows:

1. We proposed an optimal point configuration algorithm derived from the traversal methodology.
2. We proposed a method for generating random polygons, analyzing the elements that impact traversal searches, and determining the optimal traversal strides for generating models for optimal point configuration.
3. We use a convolutional neural network (CNN)-based model for point configuration and application of feature extraction and dimensionality reduction techniques on the conditions triggering configuration.

The structure of this study is as follows: In Section 2, we review the work related to this study. In Sections 3–5, we describe our proposed algorithm in detail. In Section 6, we report the simulation realization and result analysis.

## 2 Related work

There are many path planning methods, and their application ranges vary according to their own advantages and disadvantages. Based on the study of commonly used path-planning algorithms in various fields, the algorithms are classified into four categories according to the sequence of discovery and the basic principles of the algorithms: traditional algorithms, graphical algorithms, intelligent bionic algorithms, and other algorithms.

### 2.1 Traditional algorithms

Traditional path planning algorithms include simulated annealing (SA) algorithms and artificial potential field algorithms.

SA (Wang et al., 2018) algorithm is an efficient approximation algorithm for large-scale combinatorial optimization problems. It uses the neighborhood structure of the solution space to perform a stochastic search. It has the advantages of simple description, flexible use, high operation efficiency, and less restriction of initial conditions, but it has the defects of slow convergence and randomness.

The artificial potential field (Khatib, 1986; Sciavicco and Siciliano, 1988) algorithms imitate the motion of objects under gravitational repulsion and perform path optimization by establishing the gravitational field repulsive field function. The advantage is that the planned path is a smooth and safe simple description, but there is the problem of local optimization.

### 2.2 Graphical algorithms

Traditional algorithms often have the problem of difficult modeling when solving real problems, and graphical methods provide the basic method of modeling, but graphical methods generally have a lack of search capability and often need to be combined with specialized search algorithms. Graphical algorithms include C-space algorithms and grid algorithms.

C-space algorithms (Yu and Gupta, 2004) expand the obstacles as polygons in the motion space and search for the shortest path by taking the start point, the endpoint, and the feasible straight line between all the vertices of the polygons as the range of the path. The advantage of the c-space algorithm over the spatial method is that it is intuitive and easy to find the shortest path; the disadvantage is that once the start point and the goal point are changed, it is necessary to re-construct the viewable graph, which is a lack of flexibility.

Grid algorithm (Mansor and Morris, 1999) is to use encoded raster to represent the map; the raster containing obstacles is labeled as an obstacle raster, and vice versa is a free raster, which is used as the basis for path search. The Grid algorithm is generally used as an environmental modeling technique for path planning, and it is difficult to solve the problem of complex environmental information as a path planning method.

### 2.3 Intelligent bionics algorithm

When dealing with path planning problems in the case of complex dynamic environmental information, revelations from nature can often play a good role. Intelligent bionics algorithms are algorithms discovered through bionic research and commonly used ones include ant colony algorithms, neural network algorithms, particle swarm algorithms, and genetic algorithms.

Ant colony algorithm (Wu L. et al., 2023) achieves its goal by iterating to simulate the behavior of ant colony foraging. It has the advantages of good global optimization ability, intrinsic parallelism, and ease of implementation by computer, but it is computationally intensive and easy to fall into local optimal solutions, although it can be improved by adding elite ants and other methods.

Neural network algorithm (Nair and Supriya, 2020; Yu et al., 2020) is an excellent algorithm in the field of artificial intelligence, but its application in path planning is not successful because the complex and changing environment in path planning is difficult to be described by mathematical formulas. Although neural networks have excellent learning ability, poor generalization ability is its fatal flaw. However, because of its strong learning ability and good robustness, its combined application with other algorithms has become a hot research topic in the field of path planning.

Genetic Algorithm (GA) (Shao, 2021; Luan and Thinh, 2023) is an important research branch of contemporary artificial intelligence science. It is an iterative process search algorithm realized according to the principle of genetics. The biggest advantage is that it is easy to combine with other algorithms and give full play to its own iterative advantages, and the disadvantage is that the computational efficiency is not high.

Particle Swarm Optimization (Das and Jena, 2020; Zhang et al., 2020) is an iterative algorithm that simulates the behavior of birds in flight. Similar to the genetic algorithm, it starts from a random solution and iteratively searches for an optimal solution, but it has simpler rules than the genetic algorithm, and it does not have the “crossover” and “mutation” operations of the genetic algorithm. It searches for the global optimum by following the currently searched optimal value. It has the advantages of a simple algorithm, easy to implement, good robustness, not very sensitive to the size of the population, and fast convergence, but it is easy to fall into the local optimal solution.

## 3 Calculation of fraction of coverage based on polygon discretization

### 3.1 Definition of fraction of coverage

When points with Gaussian distribution are arranged in any polygon, and the measurement index is selected as the polygon fraction of coverage, the calculation result of the index is affected by the Gaussian distribution characteristics of the points, the size of the polygon, and the control range of the Gaussian points and other factors (Chen et al., 2021).

**Figure 1:**
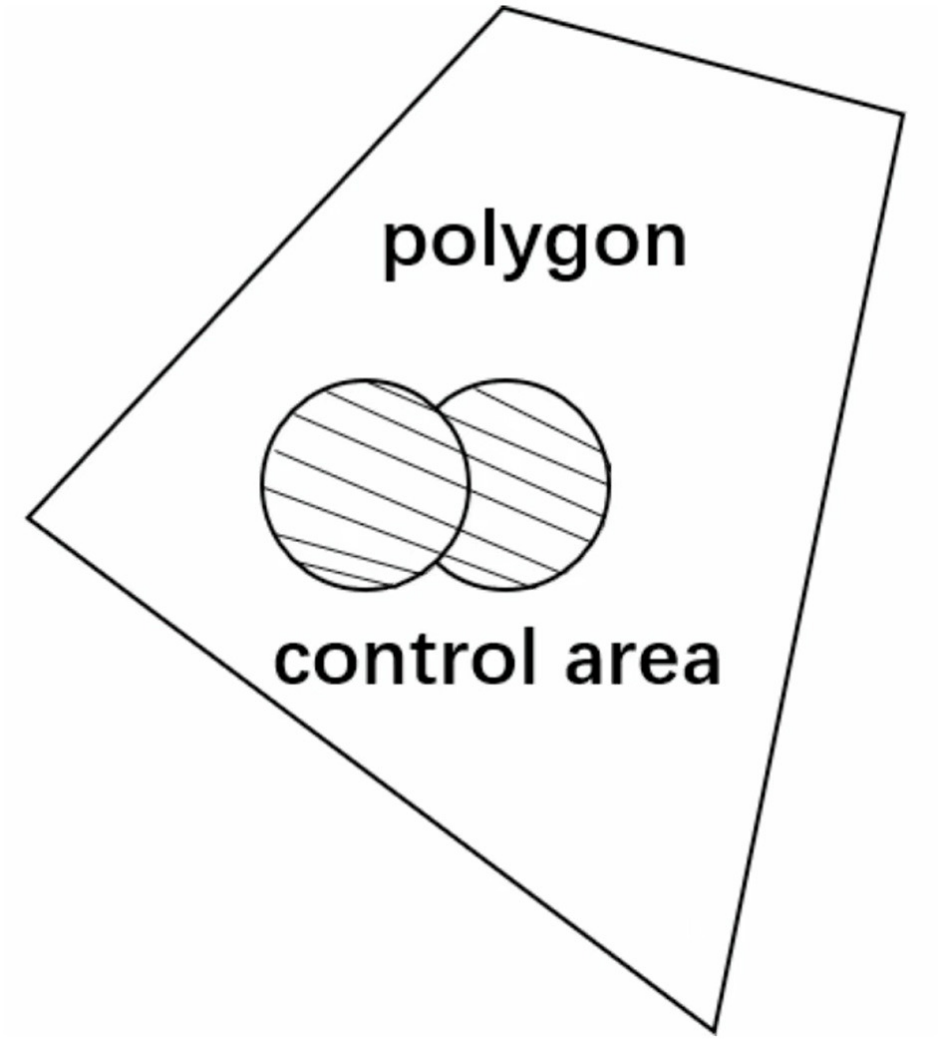
Schematic diagram of Coverage Index Calculation.

The calculation of point coverage is influenced by the randomness of the points. The method for calculating the point coverage rate is shown in Equation (1), and the joint probability density distribution function is shown in Equation (2). Given the influence of repeated coverage, it is difficult to directly solve the polygon fraction of coverage using the analytical method so that it can be calculated by discretization.


(1) $$\begin{array}{l} p=\frac{s_{f}}{s} \end{array}$$

where *s*_*f*_ refers to the point coverage, which represents the area of the intersection area between the shadow part and the polygon in the schematic diagram; s is the polygon area. The Schematic Diagram of Coverage Index Calculation is shown in Figure 1.


(2) $$f(x,z)=\frac{1}{2\pi \sigma^{2}}exp\left(\frac{{\left(x-m_{x}\right)}^{2}+{\left(z-m_{z}\right)}^{2}}{2\sigma^{2}}\right)$$

where *m*_*x*_, *m*_*z*_ represent the point configuration coordinates and σ represents the root mean square error of point Gaussian distribution.

### 3.2 Polygon discretization

For a polygon with an arbitrary shape, taking the lower left vertex as the coordinate origin and any one of the two sides connected with the change point as the *Z*-axis, a Cartesian coordinate system is established to discretize the polygon (Lei et al., 2020), and then, the coordinate calculation of any grid center point of the polygon outer envelope rectangle is shown in Equation (3).


(3) $$\{\begin{array}{c} x_{ij}=x_{\text{min}}+\frac{x_{\text{max}}-x_{\text{min}}}{n\cdot i}\\ z_{ij}=z_{\text{min}}+\frac{z_{\text{max}}-z_{\text{min}}}{m\cdot j}\\ \left(i=1,2,\ldots ,n;j=1,2,\ldots ,m\right) \end{array}$$

where *n*, *m* represent the number of discrete polygons in *x* and *z* directions; *x*_min_, *x*_max_ represent the boundary range of the polygon in the *x* direction; *z*_min_, *z*_max_ represent the boundary range of the polygon in the *z* direction.

The ray method is used to judge all the discrete points of the polygon outer envelope rectangle, in turn, and then, the polygon can be discretized (Cheng et al., 2019; Chappell et al., 2021; Siyu et al., 2022). Steps to determine whether a point is within a polygon based on the ray method:

- Step 1: Taking the center coordinate of the discrete grid point as the starting point, the ray is made along any direction, and the intersection relationship between the ray and the line segment composed of two adjacent points of the polygon is judged, in turn.
- Step 2: If the discrete grid point is on the polygon vertex, it is judged that the grid point is inside the polygon; if the ray and the line segment overlap, it is judged that the grid point is inside the polygon; regarding the odd and even case of the number of intersection points, if it is odd, the point is inside the polygon, and otherwise, the point is outside the polygon.

### 3.3 Calculation of fraction of coverage

Based on the polygon discretization, the probability of the grid is approximately replaced by the probability of the grid center point. Therefore, when n points with Gaussian distribution are arranged within any polygon, the coverage probability of the kth point for any discrete point of the polygon is calculated as shown in Equation (4), and a given point is the configuration result (Kamra and Liu, 2021), the fraction of coverage of *n* points for this grid point is calculated as shown in Equation (5).


(4) $$\{\begin{array}{c} p_{ij}^{k}=\iint_{J\leq d^{2}}f(x,z)dxdz\\ J(x,z)=(x_{ij}-x^{2})+{\left(z_{ij}-z\right)}^{2} \end{array}$$

where *d* represents the control range of the point, *x*_*ij*_ and *z*_*ij*_ denote the *x* and *z* coordinates of the discrete point, respectively.


(5) $$p_{ij}=1-\prod_{k=1}^{n}\left(1-p_{\text{ij}}^{k}\right)$$

where $p_{\text{ij}}^{k}$ represents the coverage of the *K*th point and *n* represents n points with Gaussian distribution within the polygon.

## 4 Sample generation model of optimal point configuration

### 4.1 Sample generation process of optimal point configuration

**Figure 2:**
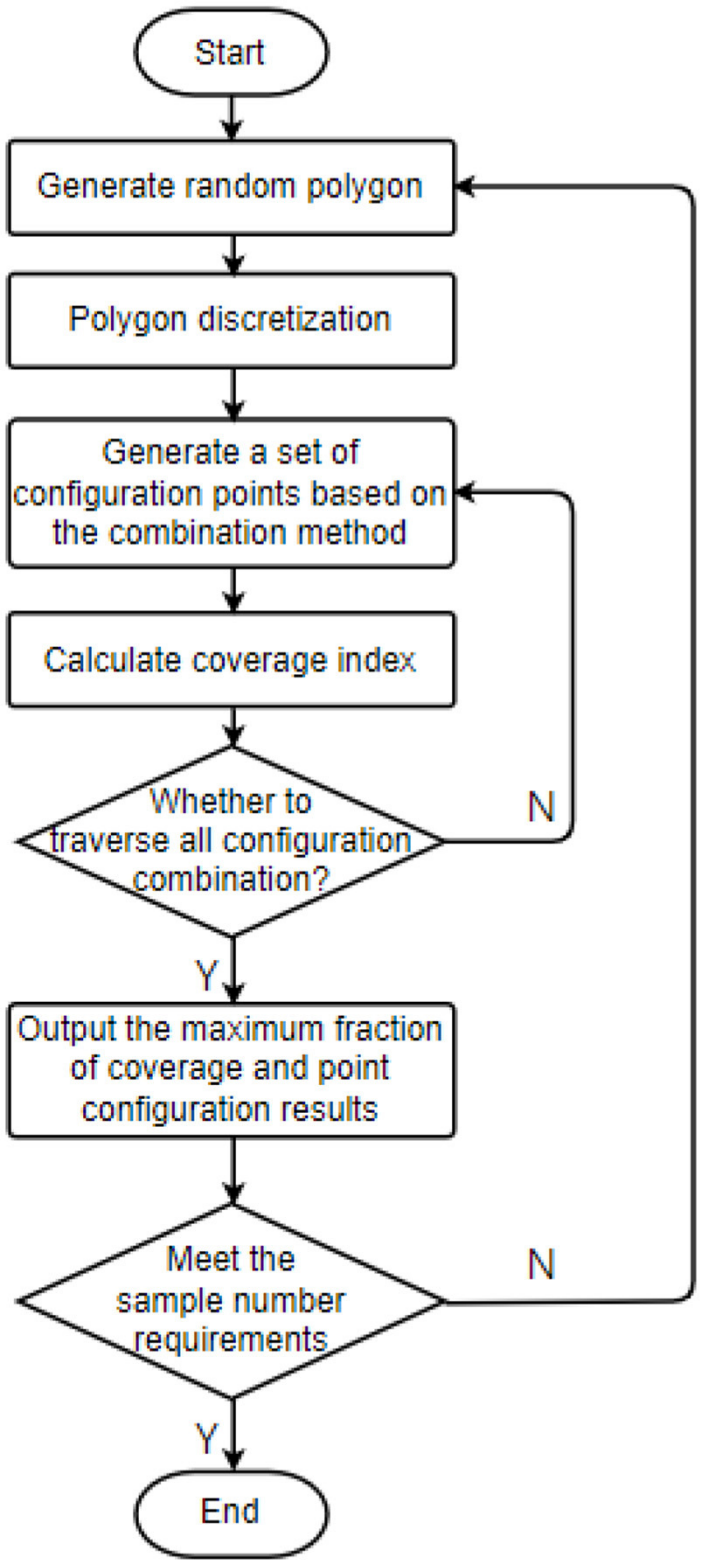
Sample generation process of point configuration.

Given the input polygons, the Gaussian distribution characteristics of the points, configuration information, and control range, we can use Equations (3–5) to calculate the fraction of coverage. The calculation is carried out by traversing all the point combinations on the inner grid points of the polygon, in turn, so that the maximum fraction of coverage and the corresponding point configuration results can be obtained; that is, a deep learning training sample can be obtained. Many training samples for point configuration can be generated by introducing variations in polygon shapes, Gaussian distribution characteristics, and control range. The sample generation process is shown in Figure 2.

### 4.2 Random polygon generation

In the region m *times* n, several random points are constructed according to the polygon edge number by using the coordinates of random function generation points (De Goes et al., 2020). Equation (6) was used to generate the coordinates of the polygon's vertices randomly.


(6) $$\{\begin{array}{c} x_{i}=m\cdot rand\\ z_{i}=n\cdot rand \end{array}$$

where rand represents a function of a random number with a probability density between [0, 1] that follows a uniform distribution.

Polygon construction: Take the point with the largest *X* coordinate as the starting point (*x*_0_, *z*_0_). If there is more than one maximum *X* coordinate, take the point with the smallest *Z* coordinate, calculate the tangent value of the included angle between the connecting line between the (*x*_0_, *z*_0_) point and other points and the horizontal line in turn, then sort by the tangent value, and label the points in turn to form a random polygon.

### 4.3 Traversal stride optimization

#### 4.3.1 Traversal stride optimization process

With the increase in the number of configuration points, the number of traversal searches increases exponentially, and the grid discretization method leads to the low efficiency of sample generation in the index calculation. While generating data sets by running code, the traversal size can be optimized according to the Gaussian distribution characteristics of points. The calculation process of Equations (4, 5) shows that the calculation of the fraction of coverage is influenced by polygon size, point control radius, and mean square error and can be changed proportionally, such as point configuration coordinates, polygon vertex coordinates, point control radius, and mean square error are all expanded by 10 times. The calculation result of a fraction of coverage is the same as the original condition. Therefore, during the optimization of the traversal stride, the outer boundary size of the polygon can be fixed, and the influence of point control radius and mean square error on traversal stride under this condition is studied, and the influence law under general conditions is extended.

In this study, when the mean square error is different, by changing the traversal stride and comparing the code running efficiency and the point configuration accuracy under different traversal strides, an appropriate stride is determined to meet the requirements of both running efficiency and accuracy within an acceptable range. The test process is as follows:

- Step 1: Randomly generate a polygon in a fixed outer boundary range *n* × *n*;
- Step 2: Take the typical control radius *R* and the typical mean square error σ; the calculation method is shown in Equation (7);

(7) $$\{\begin{array}{cc} R & =k\times \frac{n}{100}(k=1,2,\ldots ,50)\\ \sigma & =k\times \frac{n}{100}(k=1,2,\ldots ,50) \end{array}$$

- Step 3: Calculate the results of optimal configuration points under the conditions of configuring one point, two points, three points, and four points;
- Step 4: Compare the calculation results of the configuration ratio with the typical traversal stride. The calculation method is shown in Equation (8);

(8) $$\begin{array}{l} d=k\times \frac{n}{100}\left(k=1,2,\ldots ,50\right) \end{array}$$

- Step 5: Determine the optimization criterion of stride by polynomial fitting calculation and obtain the function form of Equation (9).

(9) $$\begin{array}{l} d=f\left(R,\sigma ,n_{f},number_{m}\right) \end{array}$$

#### 4.3.2 Determination of traversal stride criteria

Simulation conditions: Randomly generate an octagon in the range of 200 m × 200 m for the experiment, and the coordinates of polygon vertices are shown in Table 1.

**Table 1:** Test of coordinates of polygon vertices.

**SN**	***x***	***z***	**SN**	***x***	***z***
1	4.755	1.545	5	–38.907	18.308
2	7.598	4.822	6	–50	0.2153
3	–1.63	25.948	7	–4.646	–73.853
4	–20.572	28.315	8	24.721	–76.084

When the selected traversal strides are *d* = 0.001 n, *d* = 0.005 n, and *d* = 0.01 n, respectively, the results obtained with these parameter values are shown in Table 2.

**Table 2:** Test results under σ = 5*m* and *R* = 20*m*.

**Traversal stride**	**0.001 n**	**0.005 n**	**0.01 n**
Point configuration results	(–16, –27)	(–20, –15)	(–20, –30)
Fraction of coverage	0.2464	0.2464	0.2464
Time consumed for calculation	2.835 s	143 s	33 s

The stride determination criteria are obtained by fitting the calculation of Equation (9). In conclusion, the calculation efficiency can be improved by optimizing the traversal stride when the engineering algorithm generates data sets. A large number of data about the coordinates of polygon vertices, optimal configuration point coordinates, and the fraction of coverage can be calculated through engineering algorithms. These data can be used as annotation data of pictures for deep learning. After traversal calculation, data about polygon graphics, configuration point coordinates, and the fraction of coverage will be obtained, which will be stored and used as sample sets for deep learning model training.

## 5 Optimization model of point configuration based on deep learning

### 5.1 Feature extraction of trigger conditions

**Figure 3:**
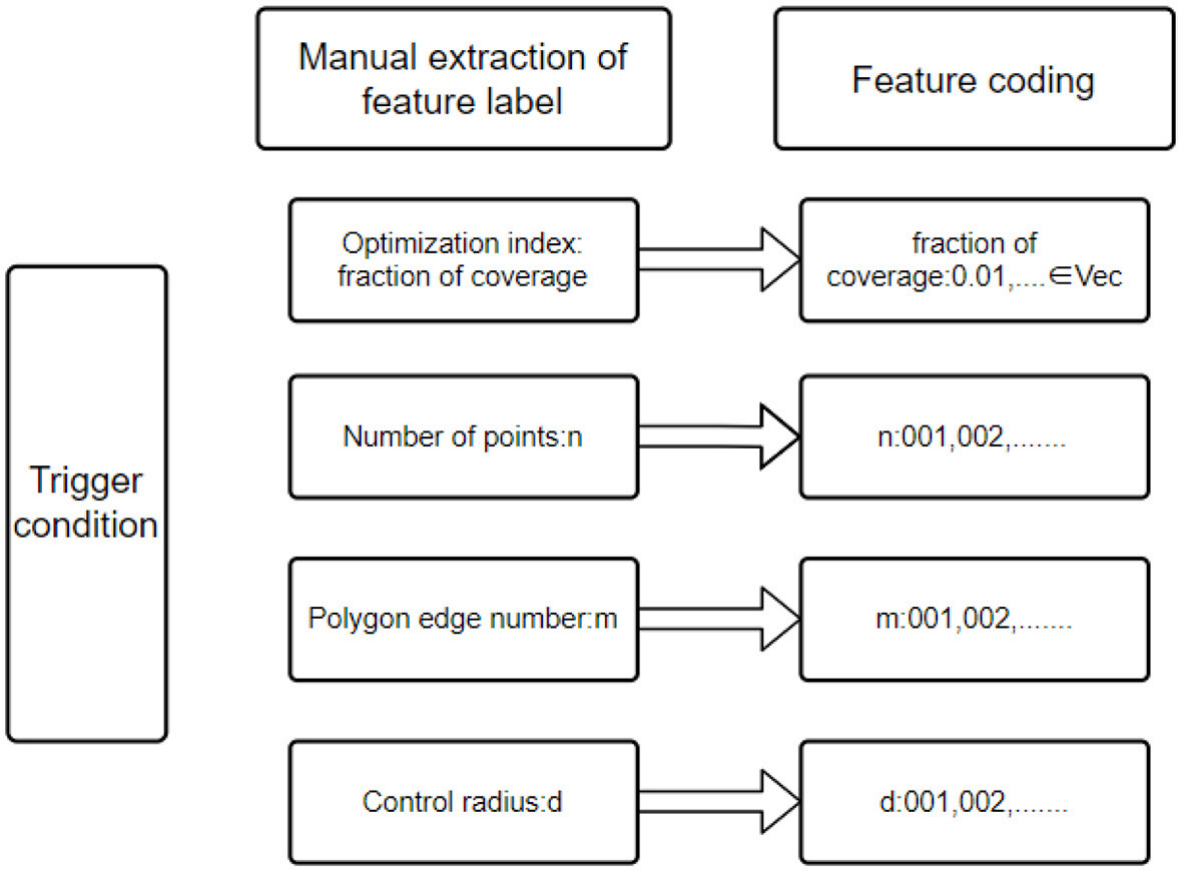
Schematic Diagram of trigger condition feature coding.

The trigger conditions mainly include fraction of coverage index, number of points, polygon edge number, Gaussian distribution characteristics of points, and control radius, as shown in Figure 3. In this study, the trigger condition features are extracted manually. The characteristic labels with practical significance are extracted according to the characteristics of the problem. The manually extracted labels can help us better understand the practical significance of the trigger conditions. To facilitate the construction of training data sets, it is the basis and premise that the training model can accurately recommend scientific and reasonable point configuration by constructing a fixed-length input vector and using the spliced feature coding as the input of the deep neural network to help the model better understand the trigger conditions of the plan (Yang and Shami, 2020).

### 5.2 Construction of point configuration model based on deep neural network

Deep neural networks are mainly divided into three categories:

- *Fully connected deep neural network* is similar to the multilayer Perceptron network. The neurons between hidden layers transmit information in the form of full connection, and the number of layers of neural networks determines the learning ability of the model (Arora et al., 2019).
- *Deep convolutional neural network* mainly deals with data with strong spatial locality and uses convolution kernel as “medium”; the output vector dimension of each layer is determined by convolution kernel, which is mainly used in image recognition, target detection, image segmentation, and other fields (Elhassouny and Smarandache, 2019).
- *Recurrent neural network* has a strong spatial correlation in processing data, mainly used in fields such as speech recognition, machine translation, and video processing (Yu et al., 2019). In view of the feature coding of the trigger, and the condition is a one-dimensional vector extracted from text data, it has no time and space correlation. Therefore, this study builds a point configuration model by the fully connected deep neural network, in which the input is the feature vector extracted from the model, the output is the grid point of point configuration, and each neural network layer in the hidden layer is composed of many neurons. The output of this model is calculated using Equation (10).

(10) $$\begin{array}{l} \text{Output}=\text{ReLU}\left(\left[\begin{array}{cccc} x_{1} & x_{2} & \cdots & x_{n} \end{array}\right]\cdot \vec{w}+\vec{b}\right) \end{array}$$

### 5.3 Polygon dimension reduction processing based on deletion point approximation method

In the actual polygon point configuration, the polygon edge number may exceed the maximum limit of feature label design (Venkateswara Reddy et al., 2022). The idea of deletion point approximation is used to reduce the polygon dimension, assuming that the vertices of the polygon are represented by (*V*_0_~*V*_*n*_), and the realization process of polygon dimension reduction is as follows:

- Step 1: Calculate the polygon area by Ear Clipping;

**Definition: Ear Tip refers to the three consecutive vertices** **V_0_**, **V_1_, and** **V_2_** **of the polygon**. If the connecting line *V*_0_, *V*_2_ is a diagonal of the polygon, *V*_1_ is an Ear Tip. **Calculation of polygon area based on Ear Clipping;**

(a) Establish a bidirectional linked list *V*_0_ of polygon vertices;

(b) Construct an initial convex vertex set **C** and a concave vertex set **R**, and construct an initial **Ear Tips set E**;

(c) **Delete one element** *V*_*i*_ **from Ear Tips set** and also delete from the vertex set in the polygon at a time, add the corresponding triangles <*V*_*i*−1_,*V*_*i*_,*V*_*i*+1_> in the triangle linked list, update the temporary vertices, and calculate whether new convex vertices and **Ear Tip** are generated;

(d) Repeat Step 3 until only three vertices remain in the linked list.

- Step 2: Delete *V*_0_, *V*_1_**, ...**,*V*_*n*_, in turn and calculate the polygon area after deleting nodes;
- Step 3: With the polygon area reduction ratio, calculated as shown in Equation (11) and sorted from small to large;

(11) $$\begin{array}{l} p=1-\frac{s_{k}}{s} \end{array}$$

where *p* represents the reduction ratio of the polygon's area, *s*_*k*_ represents the area reduction ratio for the node being considered for deletion, and s represents the initial area of the polygon.

- Step 4: Delete the node with the smallest area reduction ratio and judge whether it meets the design requirements of feature label polygons, and if so, terminate the algorithm;
- Step 5: If the condition is not met, renumber the polygon after node deletion, and then repeat steps 2–4.

## 6 Simulation realization and result analysis

### 6.1 Simulation realization

The point configuration model based on convolutional neural network mainly includes two modules: polygon shape feature extraction module and regression fitting module.

#### 6.1.1 Polygon shape feature extraction module

**Figure 4:**
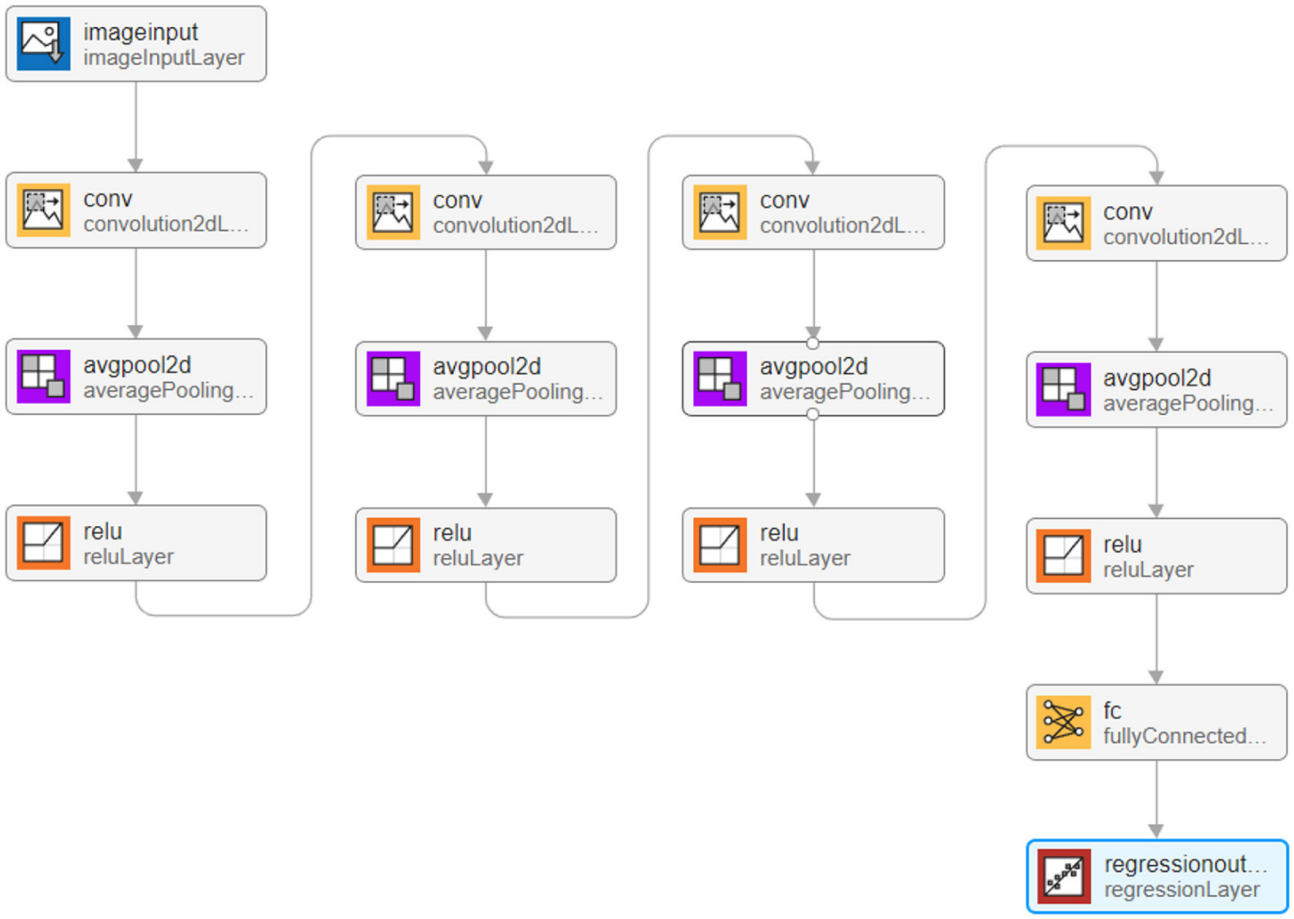
Schematic diagram of trigger condition feature coding.

The existing data sets include polygon pictures, polygon vertex coordinates, mean square error and control radius of Gaussian distribution, point configuration coordinate, and the fraction of coverage. We start by flattening the data into one dimension (it can also be flattened into 2-dimensional data, as well as 3-dimensional data, but it should always be consistent with the input layer data structure). The processed data are then fed into a convolutional layer with a convolutional kernel size of 3 × 1 channel of 16, outputting a 16-dimensional data. The BN layer and Relu activation function are then entered. Subsequent convolutional blocks operate similarly, but the dimensionality of the output is doubled. Determining the optimal number of convolutional layers and hyperparameters in our experiments is crucial for building efficient deep learning models, and given the scale of the parameters, we first determined a smaller number of network layers. We use incremental tuning of the number of network layers and hyperparameters, starting with a smaller model and gradually increasing the number of layers and tuning the hyperparameters, evaluating the performance of the model after each increase until the performance no longer improves or begins to decline. Finally, considering the influence of polygon shape on the point configuration results, four convolution layers are used to extract the characteristics of polygon shape and output the corresponding point configuration results (Zhao et al., 2021). The network structure of the polygon shape feature extraction module is shown in Figure 4.

After data processing, the image data are input into the input layer in the form of a matrix. The image is originally composed of 24-bit RGB data, and all colored images can be represented by three channel colors: red, green, and blue. Therefore, we can also achieve image matrix transformation by using the RGB of each pixel in the image. The generated image in the sample is 875 × 656 pixels. To facilitate convolution processing, the size of all images in the data set is adjusted to 510 × 510, so the input images are converted into a matrix set of 510 × 510 × 3. Take 90% of the data as the training set and the remaining 10% as the test set.

To enable the image to recognize the shape of polygons after convolution, four convolution layers are set in the deep learning model, and the parameter settings of each layer are shown in Table 3.

**Table 3:** Module 1 convolutional neural network structure diagram.

	**Number of input channels**	**Number of output channels**	**Convolution kernel**
The first convolution layer	3	32	3 × 3 × 64
The second convolution layer	32	64	3 × 3 × 128
The third convolution layer	64	128	3 × 3 × 256
The fourth convolution layer	128	256	3 × 3 × 512

ReLU is used as an activation function, and average pooling is selected in the pooling layer. The maximum number of training is 10,000, and the initial learning rate is 0.001. After four layers of convolution, *A*^1 × 4^ matrix is output after the fully connected layer, and then the point configuration result is obtained by regression analysis.

#### 6.1.2 Regression fitting module

The parameters that affect the point configuration results include not only polygon shape but also the mean square error of Gaussian distribution and control radius of points; the mean square error of Gaussian distribution and control radius of points are not extracted in the polygon shape feature extraction module. Based on this, based on the polygon shape feature extraction module, a regression fitting module is constructed to extract the mean square error of the Gaussian distribution of points and the influence of the control radius of points on the point configuration results. The input of the regression fitting module is the point configuration coordinates output by the polygon shape feature extraction module and the mean square error of the Gaussian distribution of the corresponding points and the control radius of the points. The data input size is [3,1,1], which, respectively, represents the horizontal (vertical) coordinates of the upper-level point configuration, the mean square error of Gaussian distribution, and the control radius of the point.

**Figure 5:**
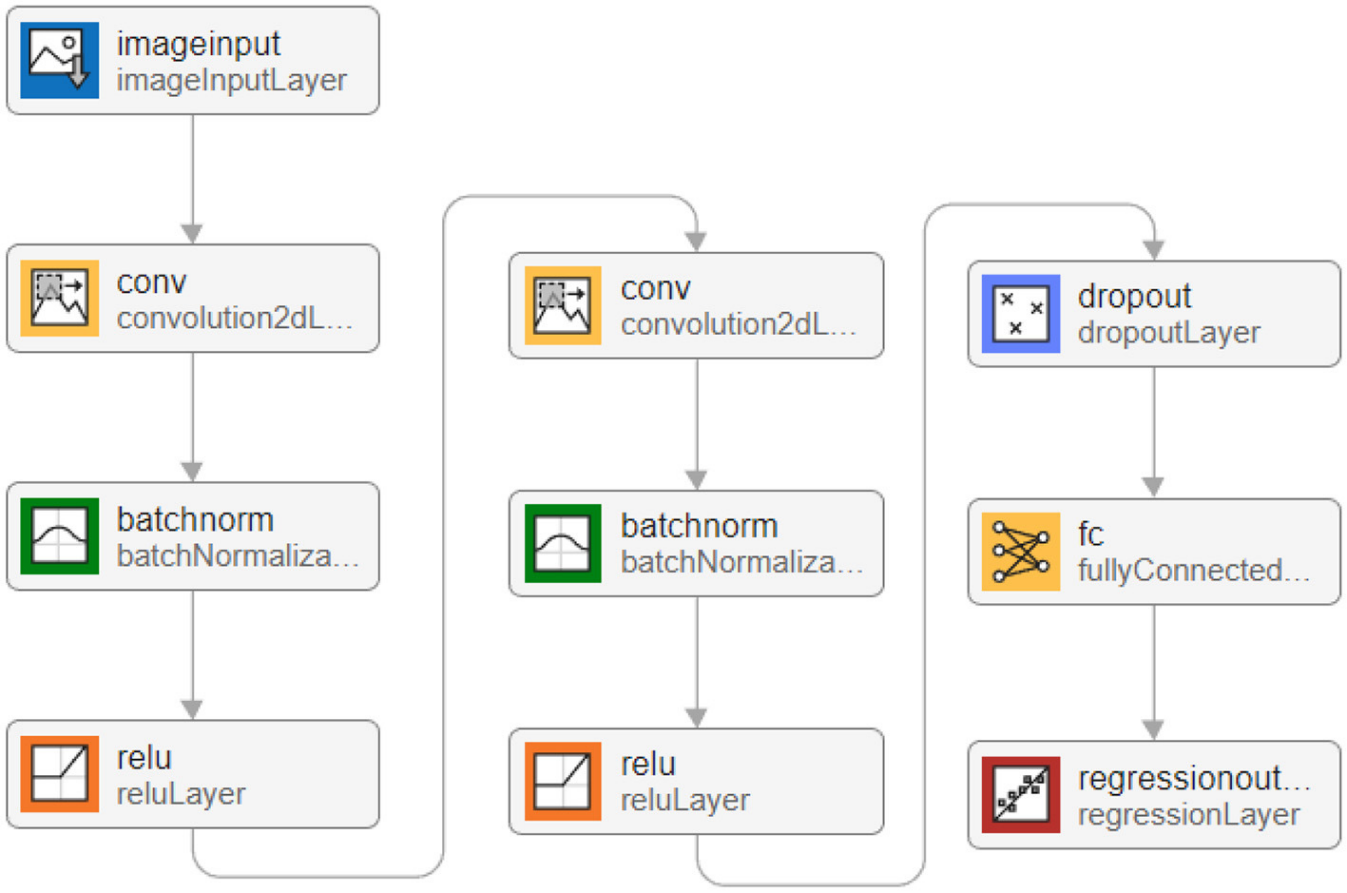
Network structure diagram of regression fitting module.

**Figure 6:**
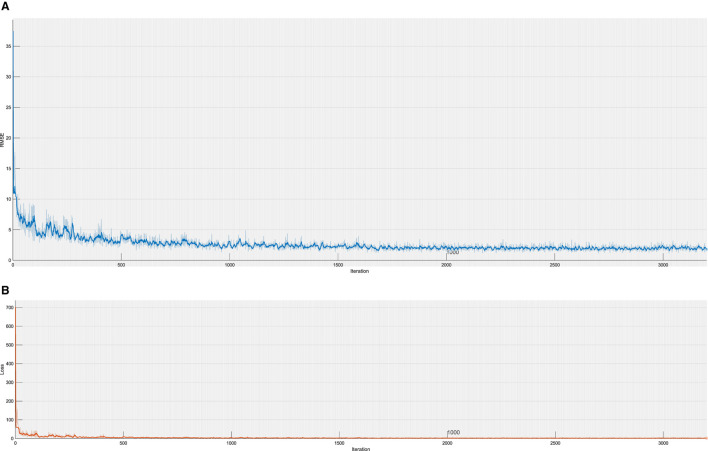
Training Convergence Process. **(A)** This is the RMSE of Training. **(B)** This is the loss of Training.

The convolution kernel size is 3 × 1, and 16 feature maps are generated by the first layer of convolution. The difference is that after the convolution layer, the BN layer is selected instead of the pooling layer to standardize the data in the convolution network. The convolution kernel size of the second layer is 3 × 1, and 32 feature maps are generated, which are also subjected to the normalization layer and Relu activation function. To prevent over-fitting, the dropout layer is set to 0.2, making it 20% possible for the activation value of neurons to stop working and making the model more generalized. SGDM gradient descent algorithm (Cui et al., 2022) is used in the parameter setting; the maximum training times is 1,600, and the initial learning rate is 0.01. Overall, 90% of the data are still used as the training set, and the remaining 10% is used as the test set. The network structure of the regression fitting module is shown in Figure 5, and the convergence process is shown in Figure 6. The results show that after training and test sets, the regression fitting gradually converges, and the training effect is good.

### 6.2 Conclusion analysis and future work

**Figure 7:**
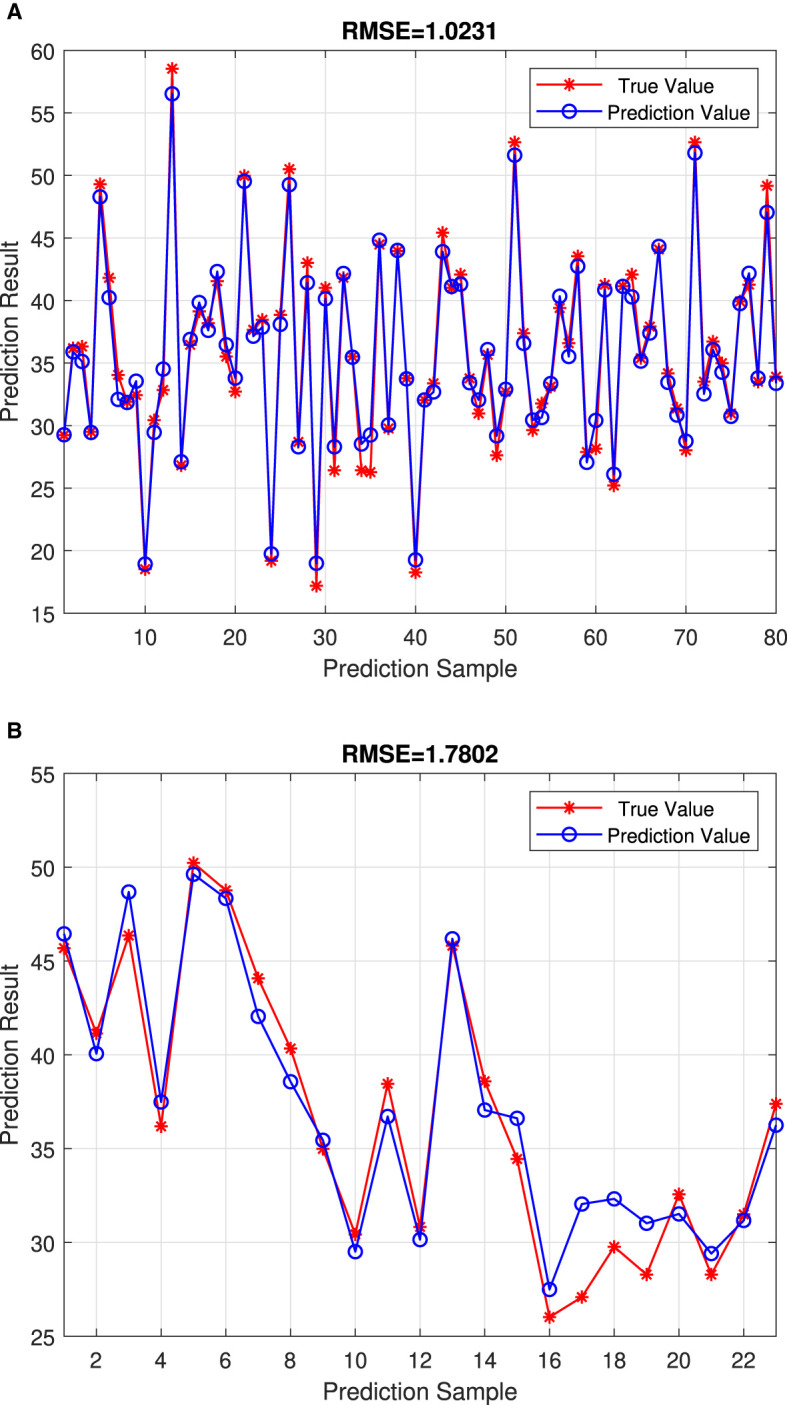
Algorithm efficiency with two-point configuration. **(A)** This is the RMSE = 1.0231 on the Training set. **(B)** This is the RMSE = 1.7802 on the Test set.

**Figure 8:**
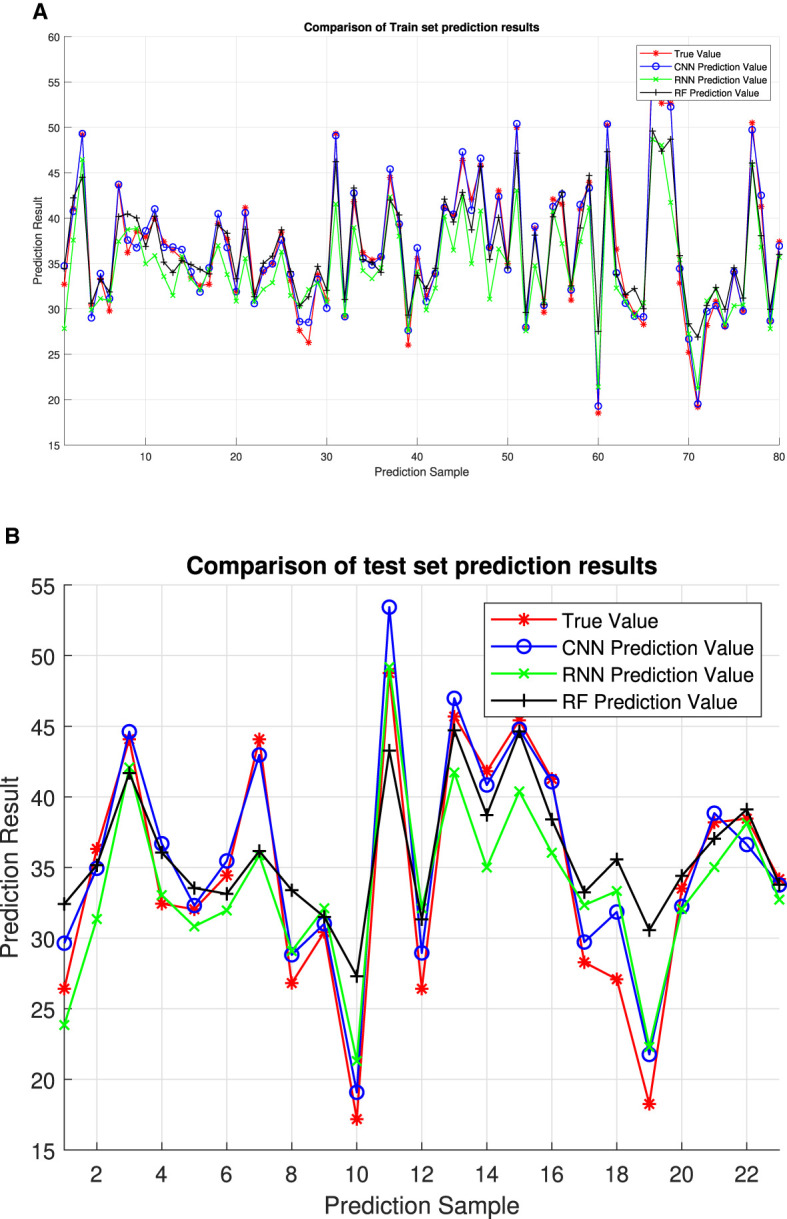
The comparison with CNN and RF. **(A)** Comparison of training set prediction result. **(B)** Comparison of test set prediction results.

This study presents a comprehensive analysis of the effectiveness and efficiency of the proposed algorithm for optimizing point configuration, which is exemplified through the optimal configuration of two points. Figure 7 illustrates the test results, showcasing the algorithm's efficacy in achieving desired configurations. Additionally, We adopted a two-point configuration and conducted a fitting regression comparison with other deep learning neural networks, Recurrent Neural Network (RNN) and Random Forest (RF). The experimental results, as shown in Figure 8, indicate that CNN's RMSE prediction performance is superior to that of RNN and RF. Table 4 presents a comparative analysis of six selected test samples, highlighting the superiority of the proposed approach. For the engineering algorithm model discussed in this study, configuring a single point with a stride of 5 m consumes ~120 s. However, the time required increases to 480 s when optimizing the configuration of two points with the same stride. In contrast, the convolutional neural network (CNN) model completes calculations within the 20 s. These results unequivocally demonstrate the superior efficiency of the CNN algorithm compared with the traversal algorithm. Importantly, the efficiency advantage of the CNN algorithm remains prominent even as the number of points increases, as it does not escalate geometrically like the traversal algorithm. Harnessing the formidable learning ability and adaptability of deep learning networks, this study extracts key features from sample data generated by traversal algorithms, training the network to generate optimal point configurations. Simulation results underscore the advantages of the deep learning-based model over traversal engineering algorithms, particularly in terms of speed and real-time performance. Moreover, the calculation index error remains within 8%, indicating the model's high accuracy and reliability.

**Table 4:** Algorithm comparison results.

**Number of targets**	**Target I**	**Target II**	**Target III**	**Target IV**	**Target V**	**Target VI**
Control radius	20	20	20	20	20	20
Mean square error	5	5	5	5	5	5
Sample point	(53, –34)	(18, –46)	(4, –43)	(2, –4)	(–27, 0)	(–7, 17)
Configuration results	(8, –39)	(–17, –16)	(–16, –3)	(–13, –1)	(8, –30)	(53, –33)
Engineering algorithm expectation	0.309937	0.274576	0.480930	0.379013	0.514694	0.656193
Time consumed for engineering algorithm	484 s	499 s	472s	480 s	508 s	492 s
Point configuration	(50, –37)	(16, –40)	(0, –40)	(5, –39)	(–27, 5)	(–9, –18)
Results based on CNN	(5, –37)	(–15, –18)	(–14, –1)	(–11, –1)	(5, –27)	(50, –30)
Fraction of coverage	0.282662	0.256812	0.463702	0.349452	0.497282	0.656193
Time consumed for CNN	21 s	20 s	21 s	20 s	20 s	20 s
Fraction of coverage error	6%	7%	4%	8%	4%	0%

In summary, this research demonstrates the transformative potential of deep learning in optimizing point configuration for robotic path planning and navigation. By significantly enhancing efficiency and accuracy while maintaining real-time performance, the proposed CNN-based approach offers a promising avenue for advancing autonomous systems in various domains. We believe that point configuration optimization algorithms can be applied in more fields. Future research directions are not limited to robot path planning, such as wireless sensor network (WSN) layout, graphics, and visual computing. In WSN layout, a point configuration optimization algorithm can be used to determine the optimal layout of sensors to maximize network coverage, extend network life, or improve data transmission efficiency. In graphics and visual computing, point configuration optimization algorithms can be applied to image reconstruction, three-dimensional modeling, animation production, and other fields to improve image quality or simulate physical phenomena by optimizing the position of points. In addition, some difficulties may be encountered in practical applications, such as the impact of complex environments, adaptability to dynamic environments, and limitations of computing resources. Therefore, we believe that the future direction of the point configuration optimization algorithm should be to introduce more advanced and efficient network models, reduce the number of parameters of the model, and improve the accuracy of the model.

## Data availability statement

The original contributions presented in the study are included in the article/supplementary material, further inquiries can be directed to the corresponding author.

## Author contributions

JW: Writing – original draft, Methodology, Conceptualization. HL: Writing – review & editing, Validation. BL: Writing – original draft, Supervision, Conceptualization. XZ: Writing – review & editing, Validation, Investigation. DZ: Writing – review & editing, Validation, Formal analysis.
